# NK Cells Promote Th-17 Mediated Corneal Barrier Disruption in Dry Eye

**DOI:** 10.1371/journal.pone.0036822

**Published:** 2012-05-08

**Authors:** Xiaobo Zhang, Eugene A. Volpe, Niral B. Gandhi, Chris S. Schaumburg, Karyn F. Siemasko, Solherny B. Pangelinan, Scott D. Kelly, Adrian C. Hayday, De-Quan Li, Michael E. Stern, Jerry Y. Niederkorn, Stephen C. Pflugfelder, Cintia S. De Paiva

**Affiliations:** 1 Department of Ophthalmology, Ocular Surface Center, Baylor College of Medicine, Houston, Texas, United States of America; 2 Department of Ophthalmology and Optometry, Wenzhou Medical College, Wenzhou, Zhejiang, China; 3 Department of Biological Sciences, Allergan Inc., Irvine, California, United States of America; 4 Department of Immunobiology, King’s College London at Guy’s Hospital, London, United Kingdom; 5 Department of Ophthalmology, University of Texas Southwestern Medical Center, Dallas, Texas, United States of America; McMaster University, Canada

## Abstract

**Background:**

The conjunctiva contains a specialized population of lymphocytes that reside in the epithelium, named intraepithelial lymphocytes (IEL).

**Methodology/Principal Findings:**

Here we characterized the IEL population prior to and after experimental desiccating stress (DS) for 5 or 10 days (DS5, DS10) and evaluated the effect of NK depletion on DS. The frequency of IELs in normal murine conjunctiva was CD3^+^CD103^+^ (∼22%), CD3^+^γδ^+^ (∼9.6%), CD3^+^NK^+^ (2%), CD3^−^NK^+^ (∼4.4%), CD3^+^CD8α (∼0.9%), and CD4 (∼0.6%). Systemic depletion of NK cells prior and during DS led to a decrease in the frequency of total and activated DCs, a decrease in T helper-17^+^ cells in the cervical lymph nodes and generation of less pathogenic CD4^+^T cells. B6.nude recipient mice of adoptively transferred CD4^+^T cells isolated from NK-depleted DS5 donor mice showed significantly less corneal barrier disruption, lower levels of IL-17A, CCL20 and MMP-3 in the cornea epithelia compared to recipients of control CD4^+^T cells.

**Conclusions/Significance:**

Taken together, these results show that the NK IELs are involved in the acute immune response to desiccation-induced dry eye by activating DC, which in turn coordinate generation of the pathogenic Th-17 response.

## Introduction

Similar to other mucosal tissues, the conjunctiva is covered with epithelium containing dendritic antigen presenting cells and a variety of intraepithelial lymphocyte (IEL) populations, lymphocytes that reside outside the lymphoid organs and in contact with epithelial cells in the gut, skin and lungs. [1] To date, several subsets of IELs have been identified in the mouse and human conjunctiva, including CD4^+^, CD8^+^, gammadelta (γδ)^+^ and NK^+^ cells. [2]–[5] The CD103 integrin has been used as a marker for IEL in different mucosal sites because it mediates homing and retention of lymphocytes to the epithelium. Its ligand, E-cadherin is highly expressed on mucosal epithelial cells. [6], [7]

γδ cells represent a small subset of T lymphocytes that have a distinct T cell receptor (TCR) that is composed of one γ-chain and one δ-chain. They are usually found in lower density than αβ T cells, and they have been implicated in maintaining tissue integrity, defending against pathogens and regulating inflammation. [1] In contrast to αβ TCR+ cells, γδ cells do not require antigen processing and MHC presentation of peptide epitopes. [8] The antigenic molecules that activate γδ cells remain largely unknown. Activated γδ cells are able to produce cytokines and exert cytotoxic effector function (by both perforin/granzyme and Fas/Fas ligand-dependent pathways). [9], [10] γδ cells have an important function in regulating immune responses, acting as gate-keepers in some tissues, by indirectly regulating cytolysis of local antigen presenting cells and epithelial cells.

Resident CD8^+^T cells have been found in the epithelium and stroma of normal human and mouse conjunctiva, [11], [12] but their function remains unknown. In non-ocular tissues, CD8^+^T cells have been found to have an immunoregulatory function. In the Lewis rat, peripheral tolerance to orally administered antigens was mediated by TGF-β secreting CD8^+^T cells. [13], [14] In the iris, CD8^+^T cells once activated in the presence of parenchymal cells, expressed and secreted enhanced amounts of TGF-β2. [15] In certain conjunctival inflammatory conditions, including graft-versus-host disease, Sjögren’s syndrome and human and experimental murine keratoconjunctivitis, a significant decrease in CD8^+^T cells with concomitant increase in CD4/CD8 ratio in the conjunctiva has been observed. [5], [11], [16] We have found that conjunctival CD8+T cells work as regulatory cells during experimental dry eye (manuscript under review).

NK cells are a subtype of lymphocytes that lack expression of the antigen receptors expressed by B and T cells; their name is derived from their ability to recognize and kill malignant cells. NKT cells are defined as NK cells that express conventional T cell receptor (TCR). Both cell types are important source of inflammatory cytokines, notably after encountering pathogens (viruses, bacteria and protozoans). NKT cells have been involved in mucosal immunity and in a variety of inflammatory/autoimmune diseases, such as experimental murine and human ulcerative colitis, asthma, multiple sclerosis and skin diseases (atopic dermatitis, psoriasis). [17]–[19]

Recently, NK cells have been implicated in both the regulation and immunopathogenesis of dry eye disease since they are an early source of IFN-γ during the induction phase of experimental dry eye disease [20]. In addition, we have recently demonstrated that NKT-derived IL-13 has a homeostatic role in maintaining conjunctival goblet cells. [21] We observed that in resting conditions, NK/NKT cells produce IL-13 and participate in the homeostatic control of goblet cell filling.

Experimental desiccating stress stimulates migration of CD4^+^T cells into the conjunctiva where they have been implicated in epithelial pathologies, including disruption of corneal barrier function and decrease in conjunctival goblet cells. [11], [22] There is consensus that the desiccating stress model of dry eye elicits both a Th-17 and Th-1 response. [20], [22], [23]

NK and NKT interaction with dendritic cells (DC) have been well documented. [24] Studies with depletion and reconstitution demonstrated that NK cells provide an early source of IFN-γ that was critical for Th-1 polarization [20], [25]; however, the effect of NK-DC interaction toward a Th-17 generation has not been explored. We have previously shown that CD4^+^T cells co-cultured with cornea and conjunctiva from DS mice required DCs to upregulate Th-17 pathway. [26] We have also shown that subconjunctival administration of liposome encapsulated clodronate, a drug used to eliminate phagoctytic cells *in vivo*, efficiently diminished resident ocular surface DCs**,** inhibited the generation of autoreactive CD4^+^ T cells, and blocked their ability to cause disease. [27]

Although known for decades that the conjunctiva harbors IEL, their function remains a black box, especially in the context of ocular surface inflammatory disease. To date, the effect of dry eye on the array of conjunctival IELs has not been investigated. We hypothesize that IEL are not bystanders, but rather active players regulating the desiccating stress-induced Th-17 immune response at mucosal sites. We also hypothesize that NK/NKT cells may switch between a “normal, protective” Th-2 tone to an “activated, pathogenic” state during desiccating stress-induced acute inflammation, ultimately driving the autoreactive Th17 response.

The purpose of this study was two fold: first to evaluate the effects of desiccating stress on the density of IEL populations and second, to evaluate the effects of depletion of NK cells on generation of the Th-17 immune response to experimental dry eye.

## Results

### The Normal Murine Conjunctiva Contains a Variety of Resident IEL

**Figure 1:**
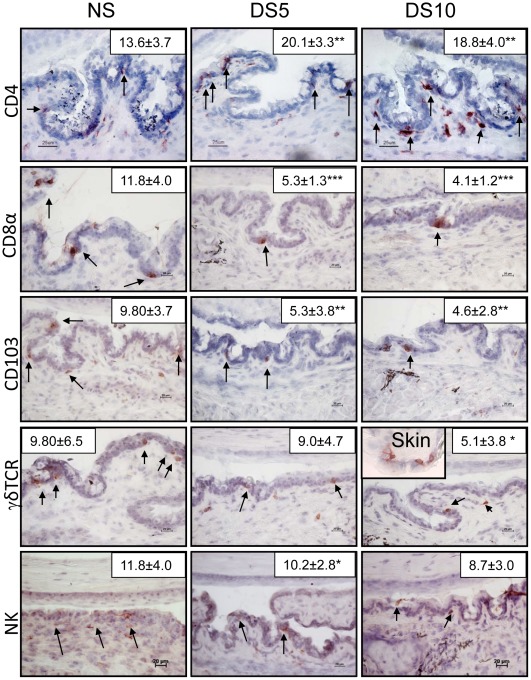
Location of intra-epithelial lymphocytes in conjunctiva before and after desiccating stress. Representative digital pictures of immunohistochemical staining of CD4^+^, CD8α^+^, CD103^+^, γδTCR^+^, NK^+^ in the conjunctivae of nonstressed (NS) C57BL/6 mice and after desiccating stress for 5 or 10 days (DS5, DS10). Inset in γδTCR row shows γδTCR in skin, which was used as positive control. Original magnification 40X; scale bar 20 µm. Number insets represent cell counts in the goblet cell rich of the conjunctiva in immunostained tissue sections in conjunctival epithelium of C57BL/6 mice. Data represents mean ± SD of cells/mm. Experiments were repeated three times with two mice per group per experiment. * indicates p<0.05, ** indicates p<0.01 and *** indicates p<0.01 comparison vs. NS control.

Here we characterized the IEL population in the normal murine conjunctiva. Sagittal eye sections from C57BL/6 mice were evaluated for the presence of immune cells (CD4^+^, CD8^+^, γδ^+^, and NK^+^ cells) and mucosal homing cell marker (CD103) by immunohistochemistry. We observed that these IEL congregate in the goblet cell rich area of the conjunctiva (Figure 1). The density of positively stained cells for these antigens in the conjunctiva epithelium is noted in Figure 1.

The number of positive cells was also quantified in freshly isolated cells from the ocular surface by flow cytometry analysis (Table 1). Splenocytes were stained using the same protocol and served as positive controls (data not shown). Lymphocyte gate was based on light-scatter properties, exclusion of double cells. Live cells in the single/lymphocyte gate was identified by propridium exclusion and confirmed by staining by CD3 marker. Flow cytometry analysis (Table 1) demonstrated that cells expressing CD103^+^ cells in normal conjunctiva were the most prevalent among the total gated CD3+ lymphocyte population (∼21%), followed by γδ^+^ (∼10%) and NKT^+^ cells (∼2.2%). CD4 and CD8α^+^T cells were the least prevalent populations identified (0.64 and 0.86%, respectively, Table 1). B220+CD3 negative cells were 4.32% of gated live cells while NK cells (CD3^−^NK1.1+ cells) were observed in similar percentage (∼4.4%).

**Table 1:** Flow cytometry analysis of freshly isolated cells from the ocular surface.

Gated from live cells	NS	DS5	DS10	P value (vs. NS control)
**B220^+^**	04.32±01.70	05.32±00.75	04.87±0.16	
**CD3^+^**	25.54±09.92	22.16±05.15	27.59±10.74	
**CD3** ^−^ **NK1.1^+^**	04.39±02.58	03.02±01.33	03.25±00.90	
				
**CD3^+^CD4^+^**	00.64±00.21	01.07±00.42	02.41±00.44	P<0.05 DS5; P<0.001 DS10
**CD3^+^CD8^+^**	00.86±00.59	01.31±00.66	03.23±00.90	P<0.001 DS10
**CD3^+^γδ^+^**	09.66±06.45	10.42±08.03	13.64±07.57	
**CD3^+^NK1.1^+^**	02.02±01.37	03.05±03.24	01.46±00.76	
**CD3^+^CD103^+^**	21.54±06.60	17.96±04.44	28.30±05.29	P<0.001 DS10
				
**Gated from live/CD3^+^ cells**				
**CD103^+^CD4^+^**	00.75±00.49	01.05±00.71	00.56±00.60	
**CD103^+^CD8^+^**	01.50±00.64	01.98±01.05	06.49±2.23	P<0.0001 DS10
**CD103^+^γδ^+^**	42.39±11.06	56.60±14.54	53.91±10.51	P<0.05 both DS5 and DS10
**CD103^+^NK1.1^+^**	02.76±01.37	06.57±03.57	02.39±01.83	P<0.01 DS5

Data is presented as mean ±standard deviation of 5–6 different experiments per group/time point. Lymphocytes were gated based on characteristic light-scatter properties (“gated cells”), subsequently gated based on forward scatter height vs. forward scatter area and propridium iodide live/dead exclusion (“live gated cells”). Data presented represents percentage of positive (^+^) or negative (^-^) cells after background subtraction.

NS = non-stressed, DS5 = desiccating stress for 5 days, DS10 = desiccating stress for 10 days.

**Figure 2:**
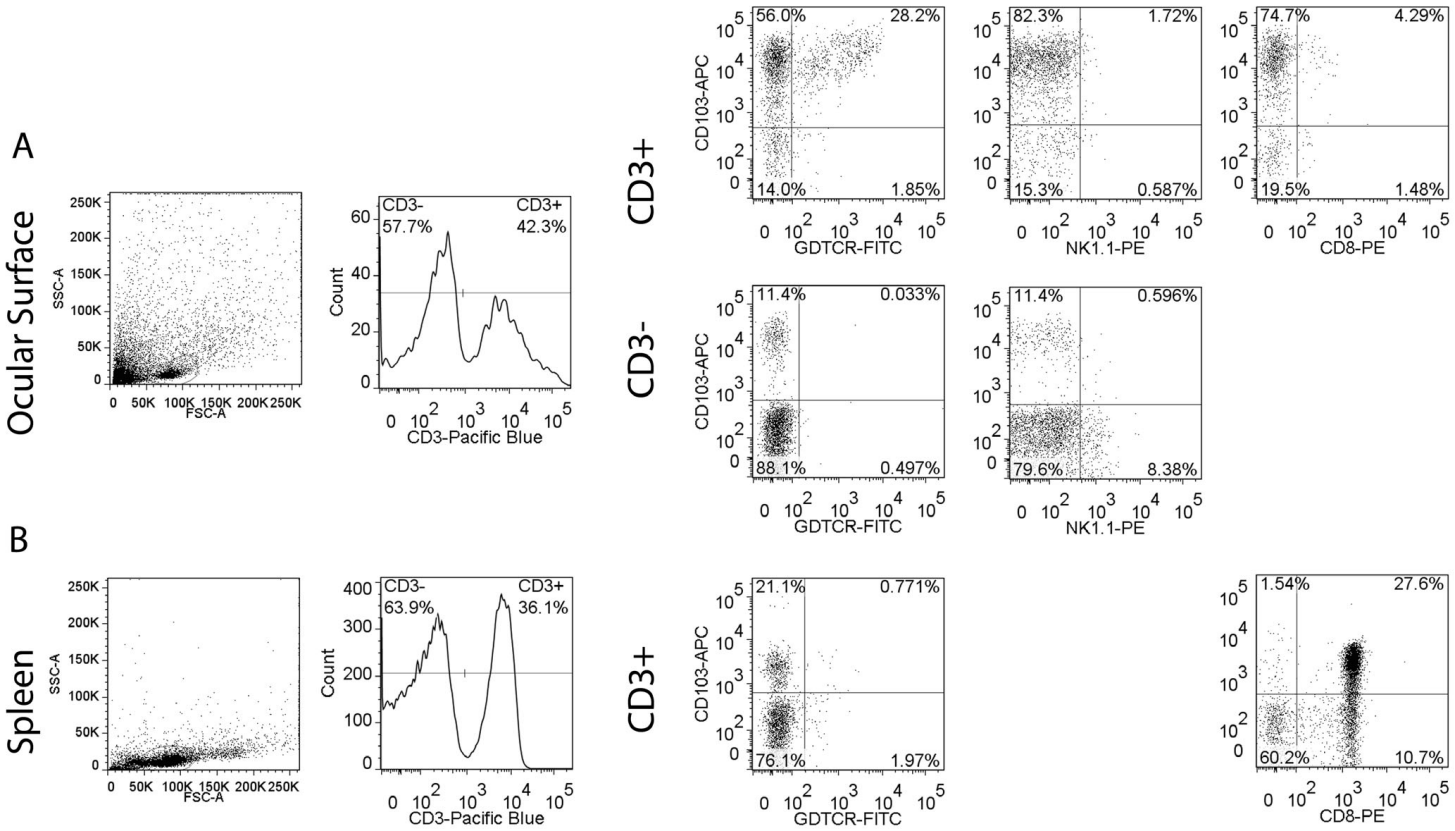
Charcterization of intraepithelial lymphocytes (IELs) in the normal murine conjunctiva. Representative flow cytometry analysis of cells isolated from ocular surface (A) or spleen (B) stained with CD3 antibody and γδ (GDTCR), CD8α, NK1.1 and CD103 markers. (+) = positive cells, (–) =  negative cells. Lymphocytes were gated based on characteristic light-scatter properties (“gated cells”, circled population on far left panels), subsequently gated based on forward scatter height vs. forward scatter area (FSC-A) and propridium iodide live/dead exclusion (“live cells”, not shown). Numbers in the quadrants indicate the percentage of cells of one representative experiment. SSC-A = side scatter area.

In the intestine, CD103^+^ IEL have been reported to have different phenotypes, mainly CD103^+^CD8α^+^ and CD103^+^CD11c^+^. [28] We observed that ∼90% of CD103+ cells were also CD3+ (Figure 2A), therefore, the phenotype of the conjunctival CD103^+^ cells was investigated using flow cytometry by dual labeling of CD103 with CD4, CD8α, NK, γδ surface markers gated from the CD3 positive population. We identified that the major population of conjunctival IEL that co-express CD103^+^ are the γδ (43%); followed by NKT (2.76%) and CD8α (1.5%), CD4 (0.75%) (Table 1 and Figure 2A). These results differ from splenocytes, where the majority of CD3+CD103^+^ was CD8α^+^ and not γδ^+^ cells (Figure 2B).

### Desiccating Stress Affects Conjunctival Intraepithelial Lymphocytes

We have previously reported that our experimental dry eye model causes an influx of pathogenic CD4^+^T cells, and a significant decrease in CD8α cells in the conjunctival epithelium when these cells are counted infiltrating the goblet cell rich area. [11] Therefore, subsets of IELs identified in the experiments described above were evaluated in mice subjected to experimental desiccating stress (DS) for 5 days and DS10. Using immunohistochemistry, we observed that the density of CD8^+^, CD103^+^, γδ^+^ cells significantly decreased in the conjunctival epithelia in response to DS, while the number of CD4^+^T cells increased as previously described [11] (Figure 1). These findings were confirmed by flow cytometry for CD 4^+^ T cells (Table 1).

### Cytokine Burst Release from NK/NKT Cells after Desiccating Stress

**Figure 3:**
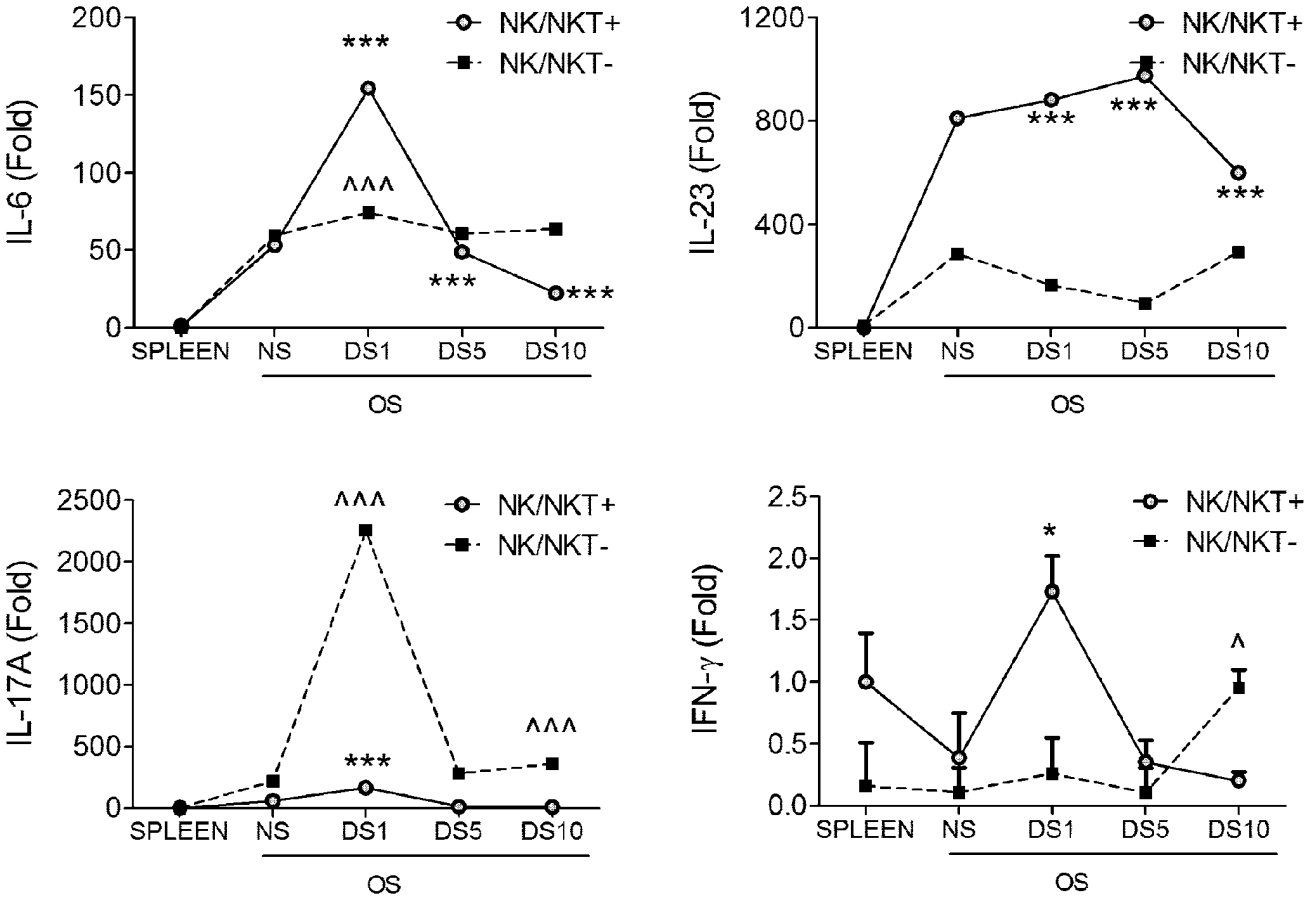
Cytokine burst from NK positive populations after DS. mRNA levels in NK/NKT positive (+) and NK/NKT negative (–) cells isolated from nonstressed (NS) spleen and ocular surface (OS) and at different time points after desiccating stress (DS; DS1 = DS for 1 day, DS5 = DS for 5 days, DS10 = DS10 for 10 days). Unfractionated spleen was used as calibrator. Experiments were repeated two times with at least three samples per group per experiment. Because the standard deviation is relatively small compared to the levels of IL-6, IL-23 and IL-17A expression, the error bars do not show in the graph. * indicates p<0.05, *** indicates p<0.001 comparison vs. NS control NK/NKT+. ^∧^ indicates P<0.05, ^∧∧∧^ indicates p<0.001 comparison vs. NS control NK/NKT−.

In our recent manuscript, we observed that NK/NKT cells produce IL-13 in normal conjunctiva that has a homeostatic role in maintaining conjunctival goblet cell mucin filling. [21] We also observed that mice chronically depleted of NK/NKT cells, as well as NKT cell deficient RAG1KO and B6.CD1dKO mice had a lower number of PAS+ goblet cells than their WT counterparts. These results point to a homeostatic role for NK/NKT cells in normal conjunctiva; however, their response to DS has not been fully investigated. To address this, we used magnetic beads to isolate NK and NKT positive and negative populations from the ocular surface (OS) before using CD49b antibody, which will identify both NK and NKT cells. We then compared the expression of Th-17 related cytokines and IFN-γ using normal splenocytes from non-stressed control mice. The mRNA levels of IL-6, IL-23 and IFN-γ were found to be significantly elevated in NK/NKT+ cells sampled 1 day after initiating DS, compared to their baseline (Figure 3). A major increase in levels of IL-17A mRNA was noted in the non-NK cells at DS1; increased expression of IL-17A was also noted in NK/NKT+ population at this time point.

These findings clearly illustrate the ability of NK to acutely respond to ocular surface DS by producing a pro-Th17 cytokine milieu.

### NK Cells Stimulate Generation of Pathogenic CD4^+^ T Cells through Activation of DCs

**Figure 4:**
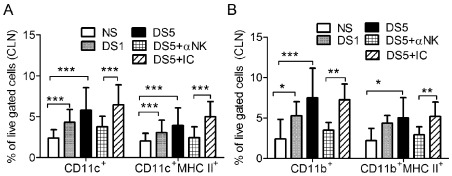
Flow cytometry analysis of dendritic cell maturation. A&B Flow cytometry analysis of CD11c^+^ and dual CD11c^+^MHC II^+^ cells (A) CD11b^+^ and dual CD11b^+^MHC II^+^ cells (B) in the CLN in non-stressed controls (NS) and after desiccating stress for (DS) for 1 (DS1) or 5 (DS5). A separate group of mice received systemic injection of depleting antibody (NK1.1) to NK and NKT cells or isotype control (IC) antibody after 5 days of desiccating stress (DS5). Data represents mean± SD. Experiments were repeated three times with at least four mice per group per experiment. *indicates p<0.05 comparison; ** indicates p<0.01 comparison, *** indicates p<0.001 comparison.

To investigate the role of NK cells in DC activation during desiccation, we performed flow cytometry analysis in freshly isolated cells from cervical lymph nodes (CLN) (Figure 4) of mice subjected for DS for 5 days, with and without NK depletion. The NK1.1 antibody that we used has been shown to both neutralize and deplete NK and NKT cells in a variety of sytems. [29]–[33] Depletion of NK1.1+ cells was confirmed by flow cytometry at the end of the experiment period (data not shown).

We observed a significant and progressive increase in the number of CD11c^+^ and CD11b^+^ cells in the draining cervical lymph node (CLN) with desiccating stress peaking at DS5. Dual labeling these cells with antibody to the murine MHC- Class II showed there was a parallel increase in DC activation (Figure 4). Depletion of NK cells prevented the increase of CD11c^+^, CD11c^+^MHC II^+^ (Figure 4A), CD11b^+^ and C D11b^+^MHC II^+^ (Figure 4B) in the CLN, indicating that NK cells can participate in DC activation.

**Figure 5:**
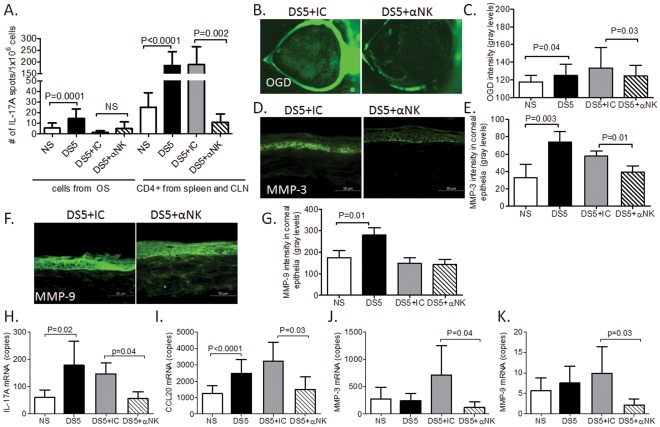
Adoptive transfer results. **A** Mean± SD of IL-17 ELISPOTs showing IL-17- producing cells isolated from the ocular surface (OS) and CD4^+^ T cells isolated from spleen and cervical lymph nodes (CLN) in donor mice that received systemic injection of depleting antibody (NK1.1) to NK and NKT cells or isotype control (IC) antibody before (non-stressed, NS) and after 5 days of desiccating stress (DS5). Experiments were repeated two times with at least five mice per group per experiment. **B** Representative images of OGD corneal staining used to generate OGD intensity score in **C.** Bar charts show mean ± SD of three independent experiments with five mice for each group per experiment. **D-G-** Laser scanning immunofluorescent confocal microscopy of cornea immunostained for MMP-3 (in **D**) and MMP-9 (in **F**) in nude mice that received CD4^+^T cells isolated from donor mice treated with systemic injection of depleting antibody (NK1.1) to NK and NKT cells or isotype control (IC) after 5 days of desiccating stress (DS5). Bar graphs are mean±SD of fluorescence intensity measured in corneal epithelium for MMP-3 (E) and MMP-9 (G) of a total of two independent experiments with at least three mice per group per experiment. **H-K**-Gene expression analyses showing mean± SD (copies) of IL-17A (in **H**), CCL20 (in **I**), matrix metalloproteinases (MMP)-3 (in **J**) and MMP-9 (in **K**) mRNA transcripts in cornea epithelia of nude mice that received CD4^+^T cells isolated from donor mice that had received systemic injection of depleting antibody (NK1.1) to NK and NKT cells or isotype control (IC) after 5 days of desiccating stress (DS5). Data represents mean ± SD. Experiments were repeated two times with at least three mice per group per experiment.

Using ELISPOT for IL-17 in freshly isolated cells from the ocular surface and CD4^+^T cells from spleens and CLN, we observed that desiccating stress for 5 days induces an increase in IL-17A+ cells in the ocular surface and in the CLN. Depletion of NK cells prior to and during desiccating stress significantly decreased generation of IL-17A-producing cells in both the ocular surface and CLN (Figure 5A).

To confirm that reduced DC activation by NK depletion would result in a biological effect due reduced generation of pathogenic Th-17 cells, we performed adoptive transfer results of CD4^+^T cells primed by DS in vivo in donors with and without NK depletion into nude mouse recipients. We and others have previously shown that IL-17A has a pathogenic role in disruption of corneal permeability that develops in response to DS. [22], [23] To confirm the effect of NK depletion in decreasing on Th-17 cells, we evaluated corneal barrier function in the nude mouse recipients using the 70 kDa fluorescent molecule OGD. As seen on Figure 5B, mice that received CD4^+^ T cells from NK depleted mice showed significant less disruption of the corneal barrier than mice receiving cells from mice treated with isotype control antibody (Figure 5B, C).

IL-17A is the “signature” cytokine of the Th-17 pathway. Th-17 committed cells produce a myriad of cytokines/chemokines, including IL-17A and CC-chemokine attractant ligand 20 (CCL20). [34], [35] IL-17A has also been shown to promote production of IL-1, tumor necrosis factor α, IL-6, IL-8 and matrix metalloproteinases (MMPs) by epithelial cells and fibroblasts. [36]–[38] We have shown that IL-17 neutralization during desiccating stress decreased immunoreactivity and expression of MMPs. [22] To evaluate if decreased number of Th-17 cell population would result in decreased MMP expression in adoptive transfer recipients, we evaluated the expression of MMP-3 and MMP-9 in corneal epithelia of these mice. Figure 5D-G shows that depletion of NK/NKT cells during desiccating stress significantly decreased immunoreactivity and gene expression for MMP-3, but not MMP-9.

Further, in support of the contribution of IL-17A in the corneal disease that develops in the nude recipients, we measured the number of IL-17A, CCL20, MMP-3 and MMP-9 mRNA transcripts in this tissue. Figure 5H-K shows that desiccating stress significantly increased the number of copies for IL-17A, CCL20 mRNA in the corneal epithelia, while depletion of NK/NKT cells significantly decreased the levels of IL-17A, CCL20, MMP-3 and MMP-9 mRNA compared to isotype control treated mice.

## Discussion

Our results show that the murine conjunctiva contains a variety of IEL populations that include γδ^+^, CD103^+^, CD8α^+^ and NK^+^ cells. The majority of CD103^+^ cells were also γδ^+^, a finding striking different from the spleen, where the majority of CD103^+^ cells are CD8α^+^. Desiccating stress had a profound effect on the resident IEL populations, causing them to decrease in frequency in the conjunctiva by immunohistochemistry. Flow cytometry analysis of live, freshly prepared cells showed an increase in CD3^+^CD8^+^ cells which may be due to better preservation of epitopes or inclusion of stromal lymphocytes in the enzyme-digested single cell suspension.

Our previous studies have shown that IL-13 derived NKT makes a significant contribution to the homeostatic maintenance of mucin filled conjunctival goblet cells. [21] In the present study, we investigated the role of NK/NKT cells in the ocular surface during the initiation and development of experimental dry eye. We showed that depletion of NK/NKT cells in mice subjected to desiccation prevented the increase of CD11c^+^ and CD11b^+^ cells in CLN and the upregulation of MHC class II, decreased generation of IL-17A-producing cells in the ocular surface and draining lymph nodes and decreased generation of pathogenic Th-17 cells.

Activated NK cells may either kill or activate DC depending on the context and the cytokine milieu. [24] The type of stimulus determines the program of DC differentiation and the type of response. Activation of a naïve T helper cell results from T cell interaction with DC. However; because three signals are required (MHC class II recognition by TCR; co-stimulation by CD28/CD80 and cytokines to direct polarization), “a three cell model” has been proposed, [39] where a third cell is the cellular source of the polarizing cytokines, since IL-4 and IFN-γ are not typical products of DC. Here we show that NK and NKT cells are rapidly activated and upregulate the levels of IL-6 and IL-23 mRNA transcripts, critical players in Th-17 pathway providing DCs with a skewed Th-17 signal milieu. Supporting our hypothesis, NKT cells were found to promote collagen-induced arthritis in DBA susceptible mice because DBA/CD1d−/− mice had less incidence and severity of disease and lower levels of IL-17A producing cells. [40]

The divergent roles of NK regulatory or pathogenic appear to be tissue-specific. Invariant NKT cells have been shown to inhibit the differentiation of CD4^+^T cells into Th-17 cells both in vitro and in vivo. [41] Oh and colleagues also showed in experimental autoimmune uveitis that uses immunization using human interphotoreceptor retinoid-binding protein peptides, CD1d−/− mice had increased disease severity compared to wild-type mice. [41] In an adoptive transfer model of colitis model, co-transfer of DX5^+^NKT cells prevented the onset of colitis or decreased the severity of established colitis. [42] Here we showed that B6 nude mice that received CD4+T cells from donor mice subjected to DS and NK depletion had ameliorated dry eye disease, better corneal barrier function and lower Th-17 related cytokines and MMPs.

Our data show that the overall percentage of CD11c+ cell expressing MHCII increases during the initiation of experimental dry eye disease and NK depletion significantly prevented it. We have previously shown that exposure to desiccating stress induces rapid production of proinflammatory cytokines and chemokines, and that acute proinflammatory cytokine production was associated with increased percentage of CD11c+ antigen presenting cells (i.e. dendritic cells) within the draining cervical lymph nodes by 24 hours post-induction of experimental dry eye [27]. Increased percentage of CD11c+MHCII+ cells correlated with an elevated percentage of CD11c+ cells bearing CD83, CD86 and CCR7. In addition, accumulation of activated APCs preceded CD4+ T cell activation (CD4+CD69+), which was observed as early as day 3 (15.1±1.0% vs. 10.5±0.5%; *p<0.001*), peaking by 6 days following exposure to desiccating stress (16.8±0.6%; *p<0.0001*). Furthermore, ablation of APCs inhibited CD4+ T cell activation and abrograted the development of experimental dry eye disease. Taken together, these data indicate that APCs are indeed activated during the initiation of experimental dry eye disease, bearing MHCII peptide, along with the appropriate co-stimulatory molecues to effectively mount an antigen-specific lymphocyte response. The importance of NK-DC interaction in other systems has been well documented but not in dry eye disease. To date, there is one report showing a significant decrease in MHC II acquisition by CD11c^+^ and CD11b^+^ cells in the CLN of NK-depleted mice. [20]

Our manuscript uses the adoptive transfer model to confirm that when CD4+ T cells are primed in vivo without the influence of NK/NKT cells, they are much less Th-17 pathogenic. There are several noveties in our study: 1) early cytokine burst release from conjunctival NK/NKT cells; 2) establishing the NK-DC link through lower acquisition of MHC II at the CLN; 3) confirming less pathogenicity of CD4+ T cells using the adoptive transfer model.

To date, there is overwhelming evidence supporting the paradigm that dry eye is an autoimmune-based inflammatory disease. The present results recapitulate the clinical signs and molecular proteins we observed when mice were subjected to desiccating stress and received an antibody anti-IL-17A. As we previously published [22] IL-17 neutralization prevented acute corneal barrier dysfunction and decreased MMP-3 and MMP-9 mRNA expression in corneal epithelium. By manipulating the NK cells during the induction phase of DS, the same results were observed. Our current findings indicate a previously unappreciated role of NK cells in the initiation and development of experimental dry eye disease. Our data demonstrate that NK cells provide a switch between a “normal, protective” Th-2 tone to an “activated, pathogenic” state acutely in response to DS that contributes to DC activation and generation of pathogenic Th-17 cells.

## Materials and Methods

### Mice

This research protocol was approved by the Baylor College of Medicine Center for Comparative Medicine (animal protocol AN-2032), and it conformed to the standards in the Association for Research in Vision and Ophthalmology Statement for the Use of Animals in Ophthalmic and Vision Research.

Female C57BL/6 (C57BL/6NTac) and B6.Cg/NTac-Foxn1^nu^NE9 (B6.nude; B6.N) were purchased from Taconic, Inc. (Germantown, NY). Mice were used at 6 to 10 weeks of age.

### Murine Desiccating Stress Model

Desiccating stress (DS) was induced by subcutaneous injection of scopolamine hydrobromide (0.5 mg/0.2 ml; Sigma-Aldrich, St. Louis), QID for 1, 5 or 10 consecutive days (DS1, DS5 and DS10, respectively). [22] Mice were placed in a cage with a perforated plastic screen on one side to allow airflow from a fan placed 6 inches in front of it for 16 h/day. Room humidity was maintained at 30–35%. Control mice were maintained in a non-stressed (NS) environment containing 50–75% relative humidity without exposure to forced air.

### Antibody-depletion of NK Cells

In a separate experiments, four group of mice were evaluated: 1) nonstressed, control mice; 2) mice subjected to DS for 5 days; 3) mice subjected to DS for 5 days that received intraperitoneal injections (IP) of anti-NK1.1 antibody [43] and 4) mice subjected to DS for 5 days that received intraperitoneal (IP) injections of mouse-IgG isotype control (Sigma-Aldrich). Mice received a total of four IP injections at days -4,-2, 0 and +2 when subjected to DS for 5 days. NK depletion was confirmed by flow cytometry in splenocytes of mice receiving antibody treatment and compared to isotype control group (data not shown).

### Isolation of Murine CD4^+^ T Cells and Adoptive Transfer

Superior CLN and spleens from donor mice were collected and meshed gently between two frosted end glass slides, as previously described. [44] Untouched CD4^+^ cells were isolated using magnetic beads according to the manufacturer’s instructions (MACS system; Miltenyi Biotec). The cells were analyzed by flow cytometry to determine T-cell purity (89% purity, data not shown). One donor-equivalent of cells was transferred intraperitoneally to T cell deficient mice. One donor-equivalent is defined as the number of cells remaining after the respective *in vitro* manipulation (e.g., CD4^+^T cells) of a single set of lymph nodes or spleen (approximately 5 × 10^6^ CD4^+^ cells). The adoptive transfer recipients were sacrificed 72 hours after the initial adoptive transfer. In some experiments, eyes were collected for histology, or were evaluated for corneal permeability while in others cornea was processed for RNA analysis. Twenty six recipient B6.N mice per group were used: five mice for histologic sections, six mice for gene analysis, and fifteen mice for corneal staining.

### Histology and Immunohistochemistry

For immunohistochemistry, eyes and anexae from each group (n = 6/time point) were excised, embedded in optimal cutting temperature (OCT) as previously published [22]. Immunohistochemistry was performed to detect and count the cells in the conjunctival epithelium and stroma that stained positively for CD4 (clone H129.9, 10 ug/mL), CD8α (clone 53e6.7, 3.125 µg/mL), γδT-cell receptor (TCR, clone GL3,10 µg/mL) [all from BD Bioscience San Diego, CA], CD103 (αE integrin, a marker of intra-epithelial lymphocytes, clone 2E7, 10 µg/mL, Biolegend, San Diego, CA) and NKp46 (a marker of NK cells [45], R&D Systems, Minneapolis, MM). Cryosections were stained with the above mentioned primary antibodies and appropriate biotinylated secondary antibodies (BD Pharmingen or; Jackson Immune Laboratories, West Grove, PA) and Vectastain Elite ABC using NovaRed reagents (Vector, Burlingame, CA), as previously described. [11] Secondary antibody alone and appropriate anti-mouse isotype (BD Biosciences) controls were also performed. Three sections separated at least 400 µm (regarding the place of cutting) per animal were examined and photographed under 10X magnification with a microscope equipped with a digital camera (Eclipse E400 with a DS-Fi1; Nikon). Individual cells that are within the epithelium or immediately adjacent of the basal membrane were counted as positive using image-analysis software (NIS Elements Software, version 3.0, BR, Nikon) as previously described. [11] Results are presented as mean ± SD of cells/mm.

### Isolation of Murine Cells

The eyes and lids of mice (n = 10/experiment, in 4 independent sets of experiments, total of 40 mice/group, in NS, DS5 and DS10 time points; n = 5 in 3 independent sets of experiments, total of 15 mice/group, in NS, DS5 and antibody or mouse IgG treated groups) were excised, pooled and incubated in 10 mL of 5 mg/mL of Dispase II (Roche Molecular Biochemicals, Indianapolis) as previously described. [22] Collected cells were used either for flow cytometry or ELISPOT.

### Flow Cytometry Analysis of Murine Cells

To characterize the IEL population, we performed two-color flow cytometric analysis using CD103, CD3, CD4, CD8α, NK and γδ specific antibodies. Single-cell suspensions of cornea and conjunctiva were stained with anti-CD16/32 (BD Pharmigen, San Diego, CA), followed by cell surface staining with FITC- anti-CD4 (clone GK1.5), Pacific-blue anti CD3 (clone 500A2) PE-CD8α (clone 53-6.7); PE-anti NK1.1 (clone PK136), FITC anti-γδTCR (clone GL3), [all antibodies from BD Bioscience] and APC-anti-CD103 (clone 2E7; Biolegend San Diego, CA). The gating strategy was: cells in the “lymphocyte” gate was identified based on light and forward scatter properties, subsequently gated based on forward scatter height vs. forward scatter area (“singlets”) and then propridium iodide live/dead exclusion (“live cells”). To characterize the phenotype of CD103+ cells, CD3+ cells were gated and the percentage of dual-labeled CD103 positive cells and one of the following antibodies: CD8α, NK1.1, CD103, CD4, γδ was noted. A BD LSRII Benchtop cytometer was used for flow cytometry in the same day and data were analyzed using BD Diva Software (BD Pharmigen) and FlowJO (TreeStar).

For evaluation of dendritic cell activation, cervical lymph node suspensions were stained with anti-CD16/32 (4°C, 10 minutes), followed by cell surface staining with anti-CD11c-FITC, anti-CD11b-APC (clone M1/70, BD Pharmingen) or anti-PE- MHC II (IAIE, clone 2G9, BD Pharmingen).

Negative controls consisted of cells stained with isotype antibody (BD Pharmigen). Cells were resuspended in violet dye (live/dead cell fixable staining, Invitrogen-Molecular Probes-Invitrogen, Carlsbad, CA) and washed, resuspended in fixation-permeabilization solution (Cytofix/Cytoperm; BD Pharmingen) and stored at 4°C until next day when analysis was performed. The gating strategy was: monocytes were identified based on forward and light scatter properties, subsequently gated based on forward scatter height vs. forward scatter area (“singlets”) and propridium iodide live/dead exclusion (“live gated cells”) followed by staining with CD11c+, CD11b and MHC II. Due to paucity of DC in the CLN, at least 200000 events were collected.

A BD LSRII Benchtop cytometer was used for flow cytometry and data were analyzed using BD Diva Software (BD Pharmigen).

### Mouse IL-17 ELISPOT

Replicate 50 uL cell suspensions containing 1.0×10^6^ freshly cells isolated from the ocular surface and CD4^+^T cells isolated from spleen and cervical lymph nodes using magnetic beads were added to 96-well PVDF plates (Millipore, Billerica, MA), precoated with anti-mouse IL-17 capture antibody (R&D Systems) as previously described. [22] The positive, red spots were counted under a dissecting microscope (SMZ 1500, Melville, NY). Replicate wells were averaged from 3 individual experiments.

### Corneal Permeability

Corneal epithelial permeability to Oregon-green-dextran (OGD; 70,000 molecular weight [MW]; Invitrogen, Eugene, OR) was assessed in fifteen mice/group in three independent experiments in B6.N recipient mice, as previously described, [22] with a minor modification. The severity of corneal OGD staining was graded in digital images by two masked observers using NIS Elements (version 3.0, Nikon, Melville, NY). The mean fluorescent intensity measured by the software inside a 2-mm diameter circle placed on the central cornea was transferred to a database and the results averaged within each group.

### Immunofluorescent Staining

Immunofluorescent staining was performed with polyclonal goat anti-MMP-3 (2 ug/mL, SC6834, Santa Cruz, CA, USA) or rabbit anti-MMP-9 (5 ug/mL, Chemicon-Billerica, MA, USA) and with appropriate secondary antibodies; Alexa-Fluor 488 conjugated IgG antibody as previously described. [22] Digital images (512×512 pixels) of representative areas of the central cornea were captured with a laser-scanning confocal microscope (LSM 510; Zeiss with krypton–argon and He–Ne laser; Carl Zeiss Meditec, Ltd Thornwood, NY) and were analyzed using the NIS Elements Software (Nikon, Melville, NY). Fluorescent intensity was measured by circumscribing the corneal epithelium of each section and the mean intensity provided by the software was transferred to a database (Excel spreadsheet, Microsoft, Redmond, WA) and the results within each group were averaged.

### NK and NKT Isolation

Single cell suspensions containing IEL were prepared as described above (n = 3/experiment, in two independent experiments in NS, DS1, DS5 and DS10; final n = 6/time point). One sample consisted for cells pooled from the ocular surface of 5 mice.

NK/NKT+ cells from ocular surface tissues from mice subjected to DS for 1 day, 5 days and 10 days and spleens from control mice were isolated using a one step procedure using magnetic microbeads directly conjugated to CD49b monoclonal antibody (MACS system; Miltenyi Biotec Inc., Auburn, CA) according to the manufacter’s instructions, as previously published. [21] Positive and negative cell populations were lysed for gene expression analysis.

### RNA Isolation and Quantitative PCR

Cornea epithelium from B6.nude recipient mice (n = 6/group) was scrapped with a scalpel; conjunctiva was surgically excised. Six samples per group were evaluated, and each sample consisted of pooled eyes of the same animal.

Total RNA was extracted using a Pico Pure RNA isolation® Kit (Arcturus, Applied Biosystems, Foster City, CA) according to the manufacturer’s instructions, quantified by a NanoDrop® ND-1000 Spectrophotometer (Thermo scientific, Wilmington, DE) and stored at −80°C. Samples were treated with DNase to eliminate genomic DNA contamination, according to the manufacturer’s instructions (Qiagen, Valencia, CA). First-strand cDNA was synthesized with random hexamers by M-MuLV reverse transcription (Ready-To-Go You-Prime First-Strand Beads; GE Healthcare, Inc., Arlington Heights, NJ), as previously described. [22]

Quantitative real-time PCR was performed with specific MGB probes (Taqman; Applied Biosystems, Inc., Foster City, CA) and PCR master mix (Taqman Gene Expression Master Mix), in a commercial thermocycling system (StepOnePlus™ Real-Time PCR System, Applied Biosystems), according to the manufacturer’s recommendations. Quantitative real time PCR was performed using gene expression assay primers and MGB probes specific for murine targets: IL-17A (Mm00439619), MMP-3 (Mm00440295), MMP-9 (Mm00442991), CCL20 (Mm00444228), IL-6 (Mm00446490), hypoxanthine guanine phosphoribosyl transferase 1 (HPRT-1, Mm 00446968, IL-23 (Mm00519942) and IFN-γ (Mm00801778).

The calculation of the copy number of the genes of interest in the adoptive transfer experiments was calculated by comparing the samples to the gene-specific standard curves, previously prepared using commercial mouse cDNA (Zyagen, San Diego, CA) while the cytokine expression in NK positive and negative populations was calculated using the comparative C_T_ method, using the unfractionated spleens as calibrator. The HPRT-1 gene was used as an endogenous reference for each reaction. A nontemplate control and total RNA without retrotranscription were included in all the experiments to evaluate PCR and DNA contamination of the reagents.

### Statistical Analysis

One way analysis of variance (ANOVA) was used to determine overall statistical significance followed by a two-tailed T-test for individual differences. P≤0.05 was considered statistically significant. These tests were performed using GraphPad Prism 5.0 software (GraphPad Software Incorporation, San Diego, CA).
